# Causal relationship between immune cell phenotypes and risk of biliary tract cancer: evidence from Mendelian randomization analysis

**DOI:** 10.3389/fimmu.2024.1430551

**Published:** 2024-07-10

**Authors:** YaLan Hu, Kui Wang, Yuhua Chen, Yongli Jin, Qiang Guo, Hui Tang

**Affiliations:** ^1^ Department of Gastroenterology, The First People’s Hospital of Yunnan Province, The Affiliated Hospital of Kunming University of Science and Technology, Kunming, Yunnan, China; ^2^ Department of Gastroenterology, Qilu Hospital of Shandong University, Jinan, Shandong, China; ^3^ The First Clinical Medical College, Lanzhou University, Lanzhou, China; ^4^ Department of Anesthesiology, Yanbian University Hospital, Yanji, China

**Keywords:** immune cell phenotypes, Mendelian randomization (MR) analysis, biliary tract cancer, causal relationship, malignant tumors

## Abstract

**Background:**

Biliary tract cancer stands as a prevalent illness, posing significant risks to human health, where immune cells are pivotal in both its development and recovery processes. Due to the diverse functionalities exhibited by different immune cell phenotypes within the organism, and the relatively limited research on their relationship with biliary tract cancer, this study employed Mendelian randomization (MR) to explore their potential association, thereby aiding in a better understanding of the causal link between immune cell phenotypes and biliary tract cancer.

**Methods:**

In this study, the causative association of 731 immunophenotype with biliary tract cancer was established using publicly accessible genome-wide association study (GWAS) genetic data through two-sample MR analysis. Sensitivity analyses assess horizontal pleiotropy and heterogeneity of the study findings.

**Results:**

Among the 731 immunophenotypes examined, a total of 26 immune cell phenotypes were found to exhibit positive results, indicating a significant association with the risk of biliary tract cancer. We confirmed that among these 26 types of immune cells, there are primarily 13 types of B cells; three types of classical dendritic cells (CDCs), including CD80 on myeloid DC, HLA DR on myeloid DC, and Myeloid DC %DC; one type of mature stage T cell,CD4RA on TD CD4+; six types of regulatory T cells; and three types of myeloid cells.

## Introduction

Biliary tract cancer (BTC) pertains to a neoplastic condition affecting the biliary system, involving the development of malignant tumors within its structures. This encompasses gallbladder cancer (GBC) and cholangiocarcinoma (CCA). Among them, CCA stands as the second most prevalent primary liver tumor worldwide, with its incidence steadily increasing over recent decades (1). Cholangiocarcinoma refers to the malignant proliferation of epithelial cells in the bile duct, forming tumors, according to its location in the bile duct, cholangiocarcinoma is classified into intrahepatic cholangiocarcinoma, hilar cholangiocarcinoma, and distal cholangiocarcinoma. Additionally, gallbladder cancer is a common malignant biliary tract cancer characterized by a mere 5% survival rate in late-stage patients (2, 3). On a global scale, the incidence of CCA varies significantly due to differences in living environments, dietary habits, geographical factors, and racial disparities. Several factors influence the incidence of biliary tract cancer, besides well-established age-related factors, these risk factors also include genetic, parasitic infections such as liver fluke, bacterial infections, smoking, alcohol consumption, dietary behaviors, and environmental factors, etc. Statistically, during the 2010–2014 timeframe, mortality rates from intrahepatic cholangiocarcinoma in men ranged between 1–2/100,000 person-years in the majority of countries (4). Due to its asymptomatic presentation, highly invasive nature, and chemotherapy resistance, the mortality rate of patients with bile duct cancer is concerning, accounting for 2% of all cancer-related deaths globally (5). In short, the mortality rate of biliary tract cancer is high, posing significant health risks, and imposing substantial financial burdens on many patients’ families. Furthermore, despite progress in therapeutic approaches, the expected outcome of individuals diagnosed with biliary tract cancer remains notably grim, rate of survival over a five-year period falls within the range of 7% to 20% (1). Currently, therapeutic modalities for BTC mainly consist of Surgical treatment, Hepatic transplantation, Chemotherapeutic treatment, radiotherapy, and immunotherapy, etc. Tumor immunotherapy, is a burgeoning field internationally, with extensive research ongoing, Immunotherapy has shown superiority over Chemotherapeutic treatment and radiotherapy. Immunotherapy is increasingly recognized as one of the most promising therapeutic strategies for advanced cancer patients (6, 7).

With the development of medicine, the role of immune cell phenotypes has emerged as pivotal in cancer treatment. In the realm of immunotherapy research, there is interest in developing patient-specific immunotherapies based on tumor-infiltrating immune cell types and their characteristics. Numerous immunotherapies, such as immune checkpoint inhibitors or cellular immunotherapy, are utilized in cancer treatment, targeting diverse immune cell populations within the immune system (8, 9). Studies targeting immunotherapy for biliary tract tumors. Increasing evidence from biological research suggests a multifaceted and close interconnection between immune cells and biliary tract cancer (10). For example, Immune therapy targets for T cells in biliary tract cancer are very attractive. Studies have shown that T cell immunotherapy targets for gallbladder cancer are attractive, with adoptive cell transfer therapy (ACT) representing a prominent approach in cancer immunotherapy (11, 12). T cells, including CD4+ and CD8+ subsets, are key players in immunotherapy.CD4+ T cells support immune responses by acting as helper T cells, aiding in the proliferation and differentiation of CD8+ T cells into cytotoxic T lymphocytes, while also boosting the phagocytic activity of macrophages. The role of tumor-infiltrating CD8+ T lymphocytes in tumor development has been examined across various human malignancies (13–15). In biliary tract cancer, cytotoxic CD8+ T lymphocytes play a crucial role and are associated with cancer prognosis. The surface MHC I molecules of biliary tract cancer tumor cells present endogenous antigens to CD8+ T cells, which in turn produce interferon-γ to attack biliary tract cancer tumor cells. Furthermore, the production of cytokines by CD4+ T lymphocytes enables indirect inhibition of tumor growth. Within the epithelial tumor infiltration in biliary tract cancer, CD4+ and CD8+ T lymphocytes exhibit synergistic anti-tumor effects (15). Studies have found that patients with biliary tract cancer who have intraepithelial tumor-infiltrating CD4+, CD8+, and Foxp3+ T lymphocytes exhibit significantly longer overall survival (16). B lymphocytes can produce various types of antibodies to recognize tumor-specific antigens and antigens associated with tumors. In the tumor microenvironment of cholangiocarcinoma, tumor-associated Immune cells, including macrophages, B cell, and T cell interact with the tumor microenvironment to inhibit tumor formation. Immune cells significantly influence the regulation of distinct biological processes in CCA, encompassing invasion, angiogenesis, lymphangiogenesis, tumor growth, and metastasis, which are also associated with the clinical prognosis of this cancer (17). These investigations into the role of immune cells in biliary tract cancer offer novel insights into potential immunotherapeutic approaches for the condition. However, the precise relationship between specific immune cell types and biliary tract cancer remains insufficiently explored. In order to propel the progression of immunotherapeutic approaches for biliary tract cancer and ameliorate patient morbidity, this investigation chose to analyze the relationship between immune cell phenotypes and biliary tract malignancies. This helps to study the pathogenesis of biliary tract cancer, provide more methods for the treatment of biliary tract malignancies, and contribute to people’s health.

Mendelian Randomization employs single nucleotide polymorphisms (SNPs) linked to exposure in publicly accessible Genome-Wide Association Study (GWAS) datasets as instrumental variables (IVs) to evaluate the potential causal link between exposure and outcome. Mendelian randomization is similar to randomized controlled trials and its analysis is rapid and inexpensive. The purpose of this method is to emulate a randomized controlled trial, thereby reducing the impact of potential confounders and reverse causation present in observational studies. And this method approach enhances causal inference regarding exposure-outcome associations (18, 19). Relatively little measurement error is associated with genetic variants and their effects. The present research employed a two-sample MR analysis to explore the causal association between immune cell characteristics and biliary tract malignancies. We used SNPs linked to immune cell phenotypes as IVs and immune cell phenotypes as exposure.

## Study design

**Figure 1:**
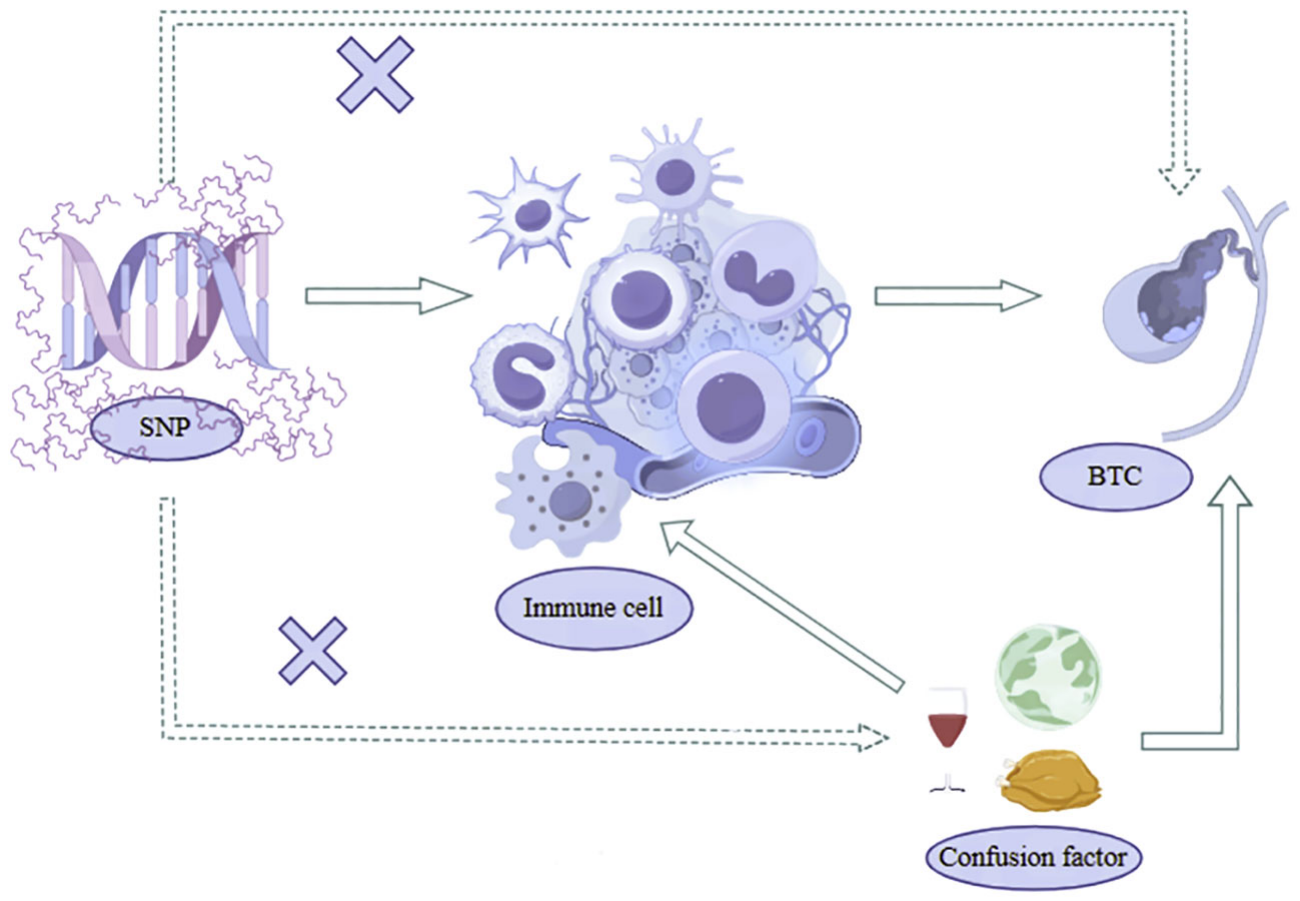
Three key assumptions of Mendelian randomization.

**Figure 2:**
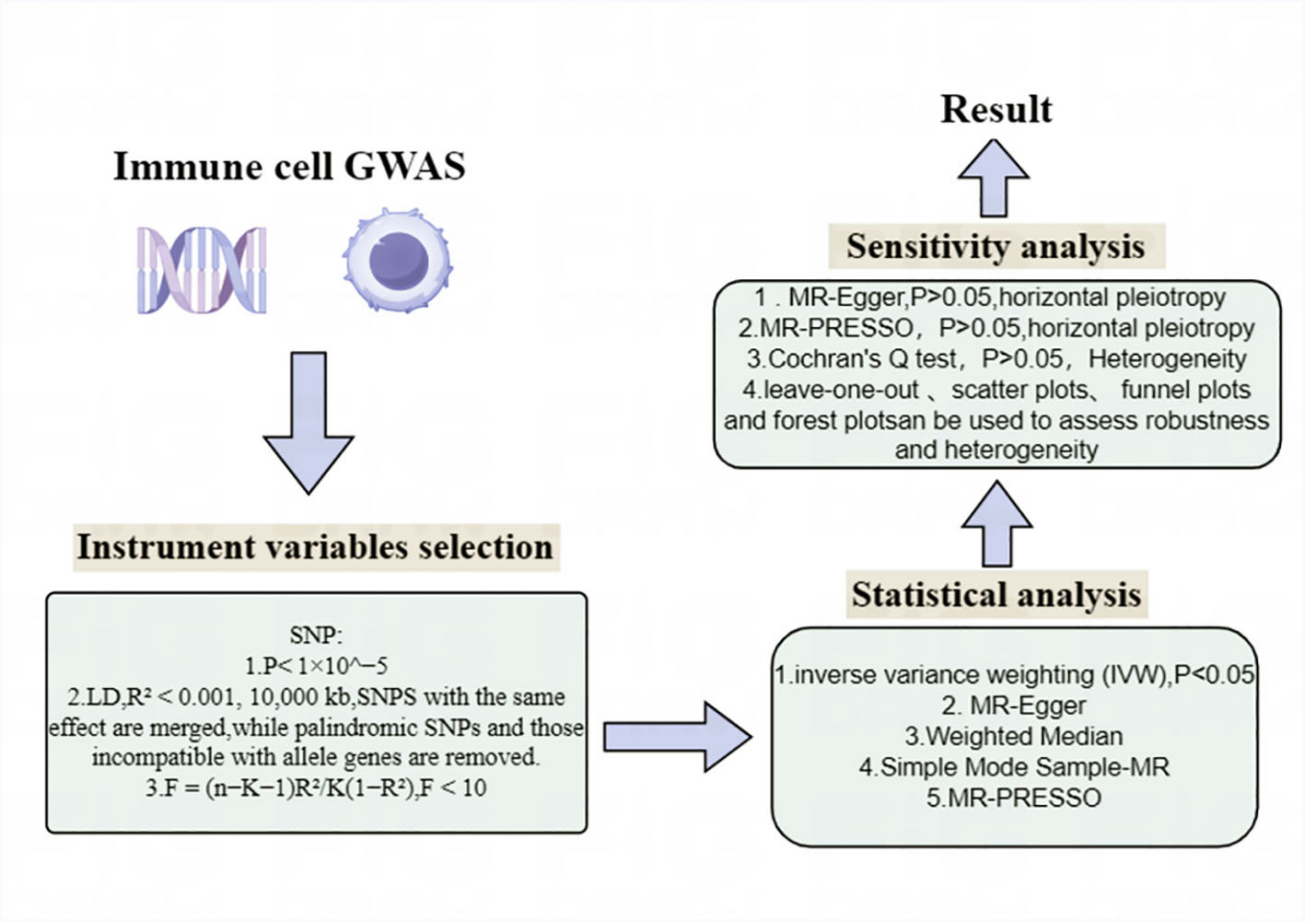
MR analysis process.

Our study utilized a two-sample Mendelian Randomization analysis to investigate the potential causal relationship between immune cell phenotypes and biliary tract cancer with SNPs strongly correlated with Immunocyte phenotypes serving as IVs, immune cell phenotype as the exposure, and biliary tract cancer as the outcome. These SNPs were identified from extensively documented Genome-Wide Association Studies literature, with 731 immune cell phenotypes obtained from published Waste SNPs adhere to three key assumptions of Mendelian Randomization analysis, as illustrated in **Figure 1**: (1) Correlation hypothesis: SNPs exhibit a pronounced correlation with he phenotypes of immune cells; (2) Independence Assumption: SNP can only affect the biliary tract cancer through the immune cell phenotypes and not through any other pathways; (3) Assuming restriction: SNPs were found to be unrelated to confounding factors such as smoking and diet (20, 21). Moreover, their impact on the outcome is exclusively mediated by their effect on the exposure, precluding any alternative causal pathways. The analysis process is shown in **Figure 2**.

## Materials and methods

### Source of data

SNPs associated with immune cells and biliary tract cancer were derived from GWAS datasets. The database GWAS used in this study is open and accessible data, and the data involved in the research were approved by the respective local ethical committees. The data originate from the FinnGen 10 local download and can be accessed at the website (https://storage.googleapis.com/finngen-public-data-r10/summary_stats/finngen_R10_C3_BILIARY_GALLBLADDER_EXALLC.gz). Accession numbers ranging from ebi-a-GCST90001391 to ebi-a-GCST90002121 were obtained from the GWAS catalog, encompassing a total of 731 immune phenotypes (**Supplementary Table S1**), Correlation analyses were conducted using a reference panel derived from Sardinian genomic sequences, based on a dataset comprising 22 million SNPs.

These 731 immune cell phenotypes encompass B cells, classical dendritic cells (CDCs), mature stages of T cells, monocytes, regulatory T cells (Tregs), myeloid cells, and TBNK cells. And these 731 immune cell phenotypes have four distinct immunological patterns:absolute cell counts (AC) encompassing 118, relative cell counts (RC) comprising 92, morphological parameters (MP) consisting of 32, and median fluorescence intensity (MFI) reflects the levels of surface antigens, with 389. Both immune cell and cholangiocarcinoma data are derived from European populations. Biliary tract cancer data include 1207 cases and 314,193 controls from European populations.

#### Instrument variables selection

For the instrumental variables(IV) used in MR, three main hypotheses are satisfied. The selection criteria for IV linked to immune cell phenotypes are as follows: The threshold for single nucleotide polymorphisms associated with cell phenotypes is set at a p-value less than 1×10^^−5^. Although a *p*-value< 5×10^^−8^ is less stringent than *p*-value< 5×10^^−8^ (22, 23), the small quantity of SNPs meeting the < 5×10^^−8^ criterion is inadequate for subsequent analyses (24, 25). In SNP selection, those with lower Minor Allele Frequency (MAF) are filtered out from the analysis due to their potentially minor impact on immune cell phenotypes, which could increase the risk of false positives. A threshold of 0.01 is set for MAF, and SNPs with MAF greater than 0.01 are retained for analysis. Additionally, by utilizing Phenoscanner for the identification of SNPs potentially linked to confounding factors, the exclusion of such SNPs serves to diminish the interference posed by these factors. This, in turn, enables a more accurate inference of causal relationships between exposure variables and outcomes. Due to the potential presence of linkage disequilibrium (LD) in SNPs, which will affect the analysis results, it is necessary to eliminate or weaken the impact of linkage disequilibrium, and remove linkage disequilibrium to obtain more precise and dependable outcomes. To attain analysis results with enhanced precision and reliability, the standard for testing LD is set at (R² < 0.001, 5000 kb) (26), where SNPs with R² greater than 0.001 within a 5000 kb range are removed. SNPS with the same effect are merged, while palindromic SNPs and those incompatible with allele genes are removed. Subsequently, each instrumental variable is evaluated using an F-test,calculating the F-statistic for each SNP.The formula for calculating F is F = (n−K−1)R²/K(1−R²), where the F-statistic threshold is used to determine IV effectiveness, set at F > 10.SNPs with F < 10 are considered weak instrumental variables and are removed.After filtering, only robust instrumental variables with F-values exceeding 10 remain, ensuring a more rigorous analysis. Finally, SNPs related to biliary tract cancer that meet the specified criteria are obtained (**Supplementary Table S2**).

### Statistical analysis

**Figure 3:**
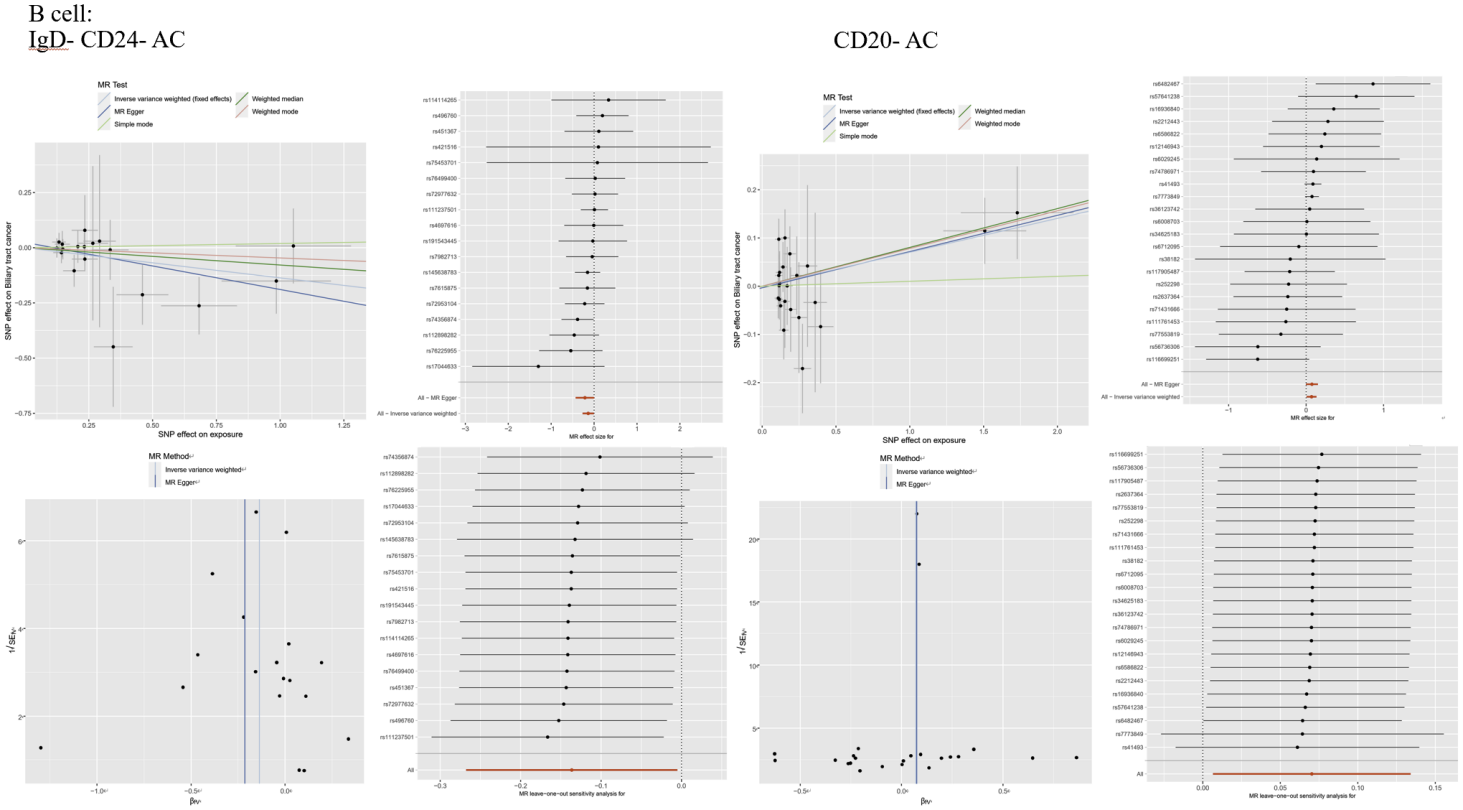
lmmunophenotype and MR Analysis of Biliary Tract Cancer: **(A)** Scatter plot, forest plot, funnel plot.and sensitivity analysis of the risk association between B cell laD- CD24- AC and biliary tractcancer. **(B)** Scatter plot, forest plot, funnel plot, and sensitivity analysis of the risk association between B cellaD-CD20- AC and biliary tract cancer.

Statistical analysis was performed utilizing R software (Version 4.2.2), augmented by the TwosampleMR package (Version 0.56), to explore the association between 731 immune cell phenotypes and biliary tract cancer via a series of Mendelian randomization analyses. This research employed various MR analysis techniques, encompassing MR-Egger, Weighted mode, Simple mode, Weighted median, and Inverse variance weighted (IVW). Among these, IVW is often preferred as the main analytical approach due to its ability to minimize the effects of confounding factors when assessing the impact of genotypes on outcome variables, thereby enhancing the accuracy of the results. For SNPs with a *P*-value exceeding 0.05 in the IVW analysis, indicating no effect, they should be excluded, leaving only SNPs with a *P*-value below 0.05. Subsequently, sensitivity analyses were conducted, primarily examining horizontal pleiotropy and heterogeneity. Horizontal pleiotropy was assessed using MR-Egger and MR-PRESSO global tests. The intercept and *P*-value from MR-Egger can be used to assess horizontal pleiotropy, where a non-zero intercept with a *P*-value less than 0.05 suggests the presence of horizontal pleiotropy. The MR-PRESSO global test is a method for detecting horizontal pleiotropy, identifying and adjusting for outliers and potential horizontal pleiotropy in MR analysis.A *P*-value less than 0.05 indicates the presence of horizontal pleiotropy, with the global test’s *P*-value also set at (*P* > 0.05). Heterogeneity was assessed using Cochran’s Q test, and the *P* value of Cochran’s Q test was set to (*P*>0.05) (**Supplementary Table S3**). The presence of outlier data points can be evaluated through scatter plots, validating horizontal pleiotropy. A sensitivity analysis, conducted by leaving out one SNP at a time, was performed to evaluate the individual impact of SNPs to the overall causal effect. Leave-one-out sensitivity analysis, funnel plots, and forest plots verify heterogeneity and robustness (**Figure 3**; **Supplementary Figure**)

## Results

### Exploration of the causal effect of immune cell phenotypes on biliary tract cancer

**Figure 4:**
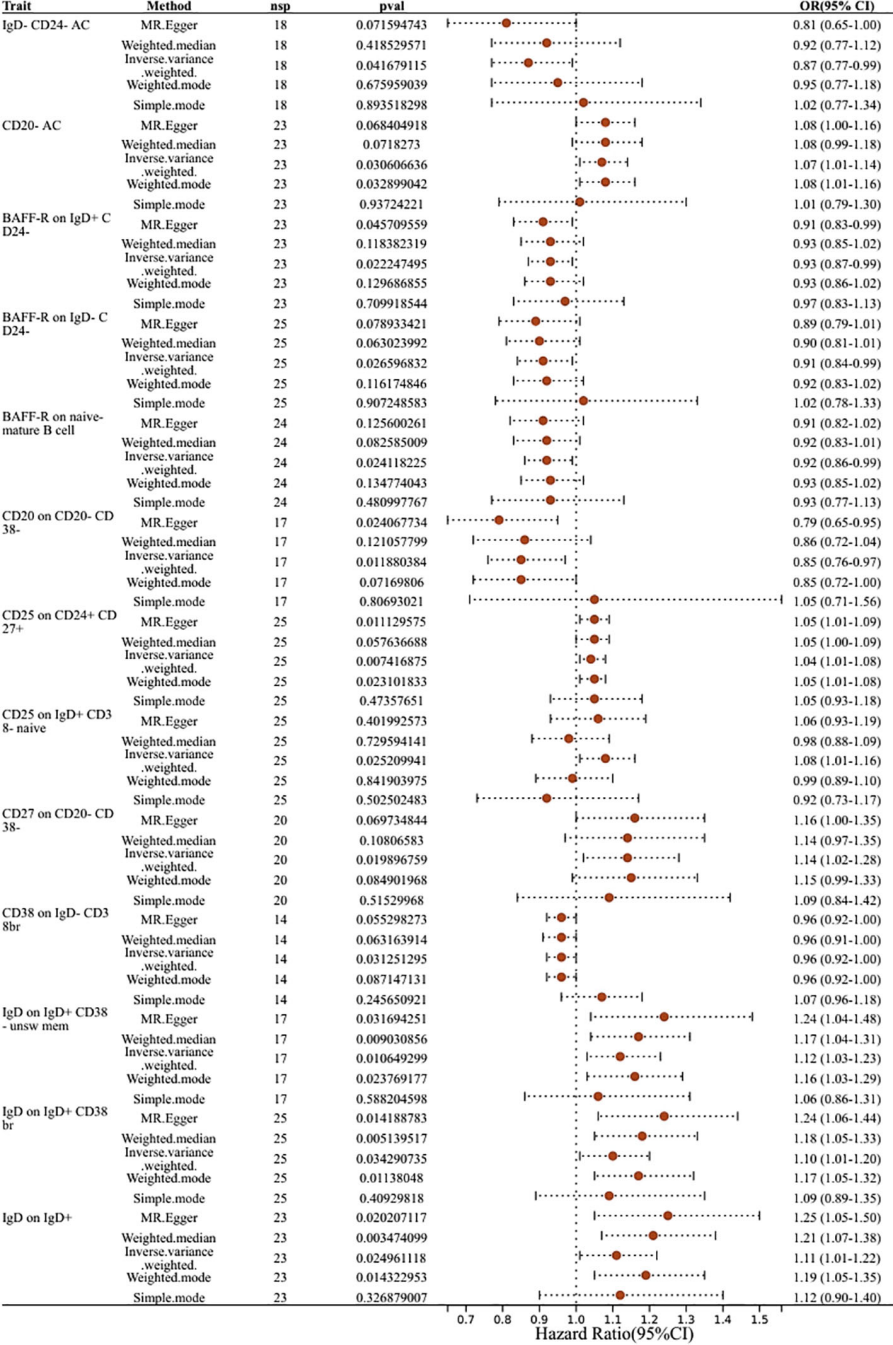
Forest plots showed the causal effect of B cell immunophenotypes on biliary tract cancer. nsnp, nonsynonymoussingle-nucleotide polymorphism; OR, odds ratio; Cl, confidence interval; P yalye, lVW analysis results P value.

This research aimed to analyze the causal link between immune cell phenotypes and biliary tract cancer. Following a comprehensive series of MR analyses on 731 immune cell phenotypes, detailed specifics for each immune cell phenotype were obtained, as outlined in **Supplementary Table S2**. Through IVW and sensitivity analyses, as well as assessments for horizontal pleiotropy and heterogeneity, and with an F-statistic > 10,26 types of immune cell phenotypes were finally positive, which means that among 731 types of immune cell phenotypes, 26 types of immune cell phenotypes were associated with biliary tract cancer. Among these, we confirmed that 13 were B cells, three were classical dendritic cells, including Myeloid DC %DC, CD80 on myeloid DC, and HLA DR on myeloid DC, one was CD4RA on TD CD4+ representing a mature stage of T cells, six were regulatory T cells, and three were myeloid cells (**Figure 4**). Details are provided in **Supplementary Table S3** (**Supplementary Tables S3, S4**).

### Causal relationship between immunophenotypes of B cells and biliary tract cancer

Thirteen immune cell phenotypes of B cells were found to be associated with biliary tract cancer. Among these,six B cell immune phenotypes exhibited positive associations with biliary tract cancer as detected by the IVW method. The IVW findings exhibited significance, consistent with the results obtained from MR-Egger, Weighted Median, and Weighted Mode analyses. Similarly, the risk estimation for biliary tract cancer by CD25 on CD24+ CD27+ was 1.04, CD25 on CD24+ CD27+ showed a significant association with biliary tract cancer risk (OR=1.04, 95% CI=1.01–1.08, *P*=0.0074). The IVW analysis of CD25 on IgD+ CD38- naive (OR=1.08, 95% CI=1.01–1.16, *P*=0.025) indicated an increased risk of biliary tract cancer. Furthermore, IVW analysis revealed positive correlations between biliary tract cancer and CD27 on CD20- CD38- (OR=1.14, 95% CI=1.02–1.28, *P*=0.0198), as well as IgD on IgD+ CD38- unsw mem (OR=1.12, 95% CI=1.03–1.23, *P*=0.01), IgD on IgD+ CD38br (OR=1.10, 95% CI=1.01–1.20, *P*=0.034), and IgD on IgD+ (OR=1.11, 95% CI=1.01–1.22, *P*=0.02). On the contrary, we identified six B cell immune phenotypes that exhibited a negative correlation with biliary tract cancer. Through IVW analysis, their respective odds ratios (ORs) were 0.87, 0.93, 0.91, 0.92, 0.85, and 0.96. The immunophenotype OR values of these immune cells were all <1, indicating that they were negatively correlated with the biliary tract cancer and had a protective effect against reduced biliary tract cancer risk. These findings were consistent across IVW analysis and other methods including MR-Egger, Weighted Median, and Weighted Mode analyses (**Figure 4**).

### Immunophenotypes of CDC and biliary tract cancer

**Figure 5:**
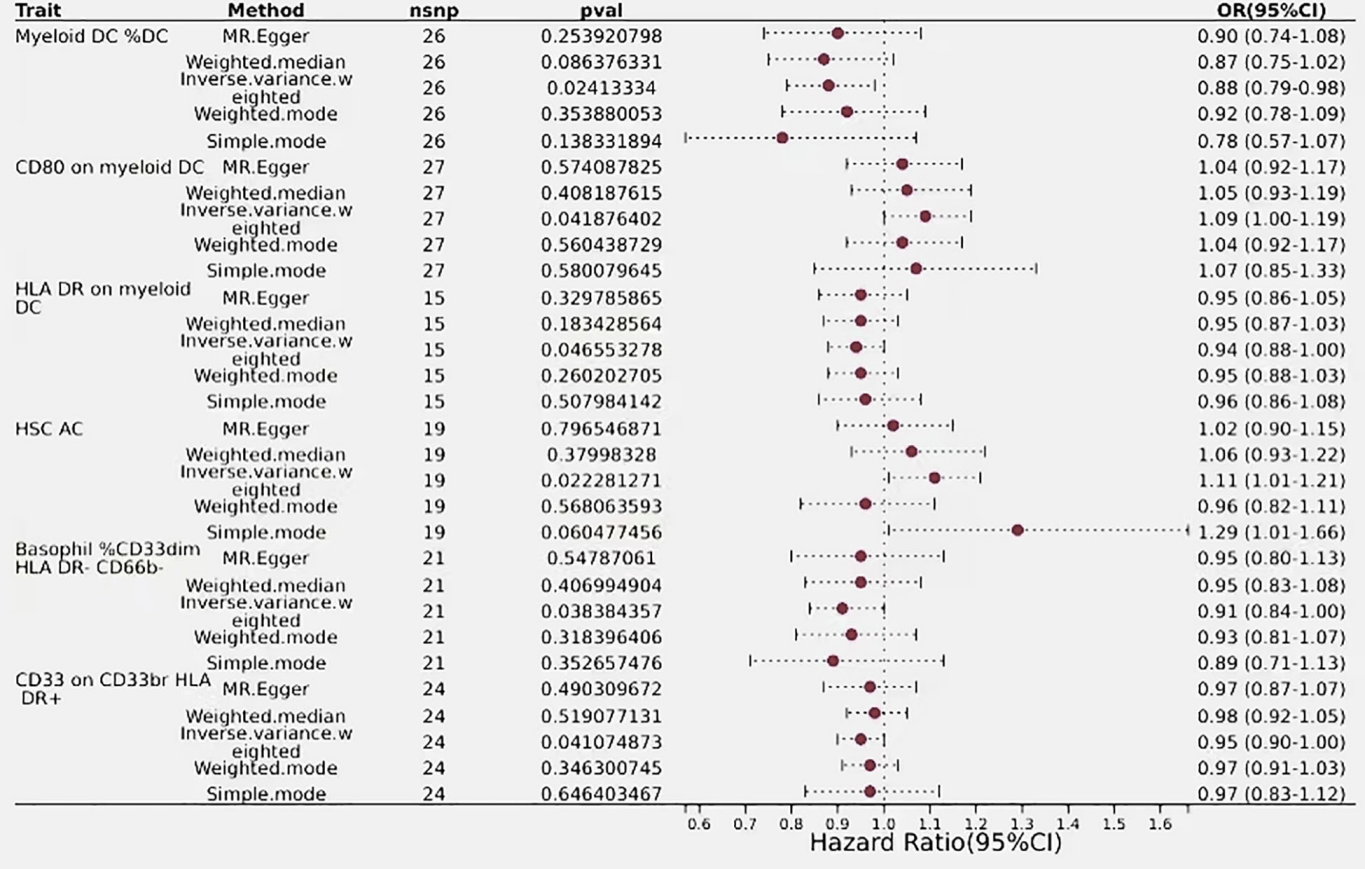
Forest plots showed the causal effect of T cells immunophenotypes on biliary tract cancer. nsnp, nonsynonymous single-nucleotide polymorphism; OR, odds ratio; Cl, confidence interval; P value, lVW analysis results P value.

The IVW analysis for classical dendritic cells revealed that CD80 on myeloid dendritic cells had an IVW result of (OR=1.09, 95% CI=1.00–1.19, *P*=0.04), indicating a positive correlation with biliary tract cancer as OR > 1 suggests. Conversely, Immune phenotypes associated with a protective effect against reduced biliary tract cancer risk include HLA DR on myeloid DC and Myeloid DC %DC. The effect size for HLA DR on myeloid DC was (OR=0.94, 95% CI=0.88–1.00, *P*=0.046), while Myeloid DC %DC was detected as (OR= 0.88, 95% CI=0.79–0.98, *P*=0.024), with OR < 1 indicating a negative correlation with biliary tract cancer. These findings regarding the phenotypes of classical dendritic cells are consistent across IVW effects and other MR analysis method (**Figure 5**).

### Immunophenotyping of T cells in relation to biliary tract cancer

**Figure 6:**
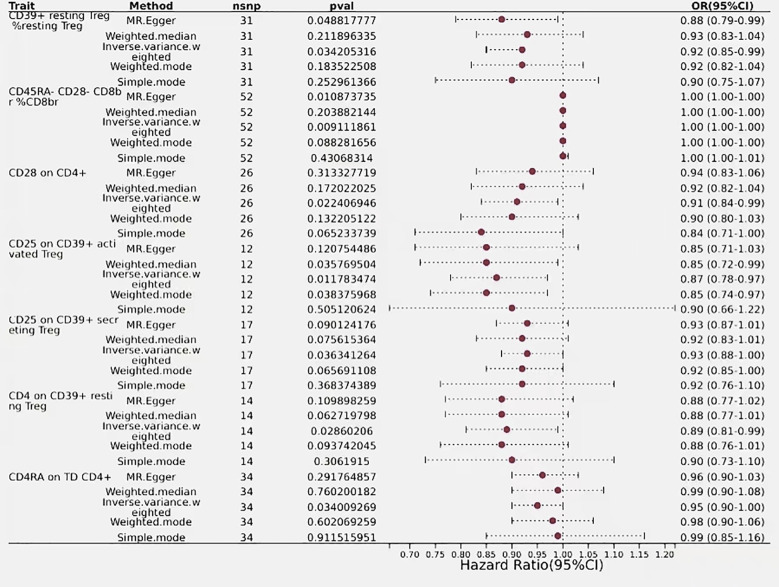
Forest plots showed the causal effect of CDC and myeloid cells immunophenotypes on biliary tract cancer, nsnp, nonsynonymous single-nucleotidepolymorphism; OR, odds ratio; Cl,confidence interval; P value, lVW analysis results P value.

In the mature stage of T cells, only one immunophenotype of T-cells in the maturation stage was negatively associated with risk of biliary tract cancer, CD4RA on TD CD4+, which was protective against a reduced risk of biliary tract cancer, as detected by an OR<1 (OR=0.95, 95% CI=0.90–1.00, *P*=0.034). Additionally, six immune phenotypes associated with regulatory T cells showed positive detection results, indicating a causal relationship with biliary tract cancer, with the majority of these phenotypes demonstrated protective properties, while only one immune trait was identified as a risk factor. The detection result for CD45RA- CD28- CD8br%CD8br was an odds ratio of 1 (95% CI=1.00–1.00, *P*=0.009), with similar outcomes observed using MR-Egger and Weighted Median,indicating that this immunophenotype has no impact on the risk of biliary tract cancer. Conversely, CD39+ resting Treg %resting Treg exhibited a negative correlation with biliary tract cancer risk, with a detection result of (OR=0.92, 95% CI=0.85–0.99, *P*=0.034), suggesting an association with an increased risk of biliary tract cancer, thus representing a risk factor for the disease. Regulatory T cells with similar outcomes included CD28 on CD4+ (OR=0.91, 95% CI=0.84–0.99, *P*=0.02), CD25 on CD39+ activated Treg (OR=0.87, 95% CI=0.78–0.97, *P*=0.01), CD25 on CD39+ secreting Treg (OR=0.93, 95% CI=0.88–1.00, *P*=0.036), and CD4 on CD39+ resting Treg (OR=0.89, 95% CI=0.81–0.99, *P*=0.028), all of which had a predictive outcome OR of less than 1 and were protective factors for a reduced risk of biliary tract cancer (**Figure 6**).

### Immunophenotype of myeloid cells in relation to biliary tract cancer

The estimated effect of HSC AC on biliary tract cancer was determined to be (OR=1.11, 95% CI=1.01–1.21, *P*=0.02), with OR > 1 indicating a positive correlation with an increased risk of biliary tract cancer, thus serving as a risk factor. Results from the Weighted Median and MR-Egger methods also support this relationship. Basophil %CD33dim HLA DR- CD66b- (OR=0.91, 95% CI=0.84–1.00, *P*=0.038) and CD33 on CD33br HLA DR+ (OR=0.95, 95% CI=0.90–1.00, *P*=0.04) from the myeloid lineage showed a negative correlation with biliary tract cancer risk, indicating an association with a decreased risk of biliary tract cancer (**Figure 5**).

## Discussion

We conducted a study to investigate the association between 731 immune cell phenotypes and biliary tract cancer. Through a series of MR analyses following strict criteria, we pinpointed 26 Immunocyte phenotypes correlation with biliary tract cancer. This is also the first study to explore the genetic contributions of these 26 immune cell phenotypes to biliary tract cancer.

In tumors, T lymphocytes and B lymphocytes exert inhibitory effects on tumor progression, which correlates with the prognosis of biliary tract malignancies patients. Within the framework of biliary tract cancer, tumors infiltrated by immune cells trigger an immune response (27, 28), and these immune cells infiltrating the tumor include T cells, B cells, myeloid cells, natural killer(NK) cells, dendritic cells, and macrophages, which interact with each other to generate an immune response to inhibit tumor progression. Tumor-infiltrating lymphocytes (TILs) play a pivotal role in fostering anti-tumor immune responses by detecting tumor antigens and executing the destruction of tumor cells (29). Among these immune cells infiltrating the tumor, T cells and B cells are the primary cells involved in anti-tumor activity and are also the main cells generating immune responses. B cells have the capability to produce antibodies and participate in immune responses through various mechanisms. This research indicates a significant negative relationship between CD20 on expression of CD20 on B cells and the risk of biliary tract cancer. The results indicate that elevated levels of CD20 on B cells could potentially exert a protective influence against the onset of biliary tract cancer. Within the phenotype of immune cells expressing CD20 on CD20- CD38-, B cells exert a protective effect against biliary tract cancer risk, whereas cells lacking CD20 expression such as CD20- AC and CD27 on CD20- CD38- promote the risk of biliary tract cancer. CD20, a surface molecule on B cells, is expressed at almost every stage of B cell development. Its expression is crucial for aiding in the development, maturation, and activation of B cells, playing a vital role in the regulation of B cell functions. CD20 is not known to have a natural ligand. Its primary function is to enhance B cell immune responses, playing a pivotal part in the initiation of T cell independent antibody responses (30). Kasper et al. found a significant increase in CD20+ B cell abundance within the TILs of Intrahepatic cholangiocarcinoma (ICC) patients infected with EB virus, the heightened density of CD20+ B cells correlates significantly with extended survival rates in ICC (31). Additionally, upon binding with antibodies, CD20 is capable of generating signals that regulate cell proliferation and programmed cell death in diverse cell types, including neoplastic cells (32). It is noteworthy that anti-CD20 therapies are applicable across various diseases, with several pharmaceutical companies investing in robust research on CD20. CD20 antibodies currently represent one of the most successful anti-tumor treatment strategies. Moreover, the heightened occurrence of CD20+ B cells shows a positive correlation with the clinical outcomes of patients afflicted with breast cancer, melanoma, colon cancer, and biliary tract cancer (33). These research elucidate the role of CD20 in inhibiting the progression of biliary tract cancer, consistent with our research findings predicting a correlation between. The results of the current study show that BAFF-R on IgD+ CD24-, BAFF-R on IgD- CD24-, and BAFF-R on naive-mature B cells are inversely associated with biliary tract cancer, contributing to the prognosis of patients with this condition. Conversely, the expression of CD24 on CD25+ CD24+ CD27+ B cells is positively correlated with biliary tract cancer, confirming CD24 as a risk factor and BAFF-R (B-cell activating factor receptor) as a protective factor.BAFF-R is a surface receptor found on B cells that specifically binds to BAFF. This interaction fosters the viability, proliferation, and differentiation of B cells. BAFF transmits co-stimulatory cues to T cells, fostering inflammation through the Th1/Th17 pathways (34). BAFF functions as a co-stimulatory signal that assists in the activation of both naïve and memory CD4 and CD8 T cells (35–37). CD4 and CD8 T cells are pivotal in orchestrating anti-tumor immunity in the biliary tract cancer microenvironment (38–40). These findings underscore the importance of the interaction between BAFF-R on the surface of B cells and BAFF for their normal function and immune responses, particularly in tumor immunity. BAFF-R emerges as a protective factor in reducing the risk of biliary tract cancer. Tumor-initiating cells (CSCs), alternatively termed as tumor stem cells,exhibit drug-resistant, can metastasize and spread, which is the key to tumor development, metastasis as well as recurrence. And CD24 is one of the markers expressed by tumor stem cells. Evidence suggests that 20%-30% of CCA tumors express cancer stem cell markers, heightening the risk of tumor progression and recurrence among patients with unfavorable prognoses (41). Furthermore, CD24 overexpression in primary gallbladder carcinoma (GBC) correlates with Lymph node spread and invasion into lymphatic and vascular channels, and the worst prognosis was observed in patients with primary gallbladder carcinoma who were in the CD24+ subgroup (42). Studies have indicated that CD24 serves as a notable indicator of malignancy and predictor of poor outcomes in GBC (43). These studies are consistent with our analysis, demonstrating that CD24 is a risk factor for biliary tract cancer.Additionally, research has revealed a positive correlation between CD25 on IgD+ CD38- naive B cells and biliary tract cancer.

The detection of CD4RA on TD CD4+ within mature T cells showed an inverse link with the risk of cholangiocarcinoma. Specifically, it is the CD4+ T cells that express CD4 during the mature stage. This study implies an association between CD4RA+CD4+ T cells and a decreased risk of biliary tract cancer. In addition to promoting clonal expansion, CD4 T cells are also involved in the differentiation of CD8 T cells into effectors and effector/memory cells (44). Activated CD4 T cells provide not only CD40L, but also IFN-I, which act nonredundantly to cross-prime cDC1s against tumor cell-associated antigens (45). A follow-up investigation involving 306 individuals diagnosed with biliary tract cancers demonstrated a positive correlation between longer overall survival (OS) and increased tumor infiltration of total CD4+ tumor-infiltrating lymphocytes (46). Helper T cells that express CD4RA+CD4+ acquire the capability to recognize CD4 antigen peptides consisting of amino acid residues. Upon activation, naive CD4RA+CD4+ T cells can differentiate into different types of Th cells (e.g., Th1, Th2, Th17), which can present antigens and secrete cytokines to promote inflammation and promote the proliferation. Moreover, they have the capacity to stimulate CD8+ T cells, guiding them toward targeted tumor cell destruction, while also fostering B cell differentiation, thereby enhancing the immune response. They exhibit a suppressive effect on the progression of biliary tract malignancies. The immunophenotypes of regulatory T cells, including CD28 on CD4+, CD4 on CD39+ resting Treg, CD4 on CD39+ resting Treg, and CD39+ resting Treg % resting Treg, CD25 on CD39+ activated Treg, exhibit a significant causal relationship with biliary tract cancer, serving as protective factors against its development. This underscores the association between CD4-expressing regulatory T cells and biliary tract cancer. Biliary tract cancer typically arises from conditions such as gallstones, pancreaticobiliary maljunction (PBM), or infrequently, gallbladder polyps. These malignancies are frequently associated with chronic inflammation (47). CD4+ Tregs are crucial in inflammation-linked diseases, exerting a suppressive impact on inflammatory responses within the tumor microenvironment (TME) (48–50). In addition, studies have shown that CD39 Tregs efficiently suppress T cell proliferation and the secretion of inflammatory cytokines like interferon (IFN)-γ and interleukin (IL)-17 (51, 52). In the context of biliary tract cancer inflammation, immune cells may mitigate immune inflammation, thereby potentially reducing tumor growth and metastatic potential within the tumor microenvironment.Thus, the increased expression of CD4 or CD39 regulatory T cells contributes to dampening inflammation in biliary tract cancer.

Our study determined that myeloid DC %DC and myeloid dendritic cell HLA DR were protective factors for biliary tract cancer.Myeloid dendritic cells assume a central role in the process of antigen presentation, wherein they capture, process, and present antigens to T cells, thereby instigating and coordinating adaptive immune responses.Moreover, myeloid DCs participate in the activation and differentiation of diverse subsets of immune cells. they endowed with the remarkable ability to activate both cytotoxic CD8+ T cells and helper CD4+ T cells (53–55). In patients with biliary tract cancer, those with dendritic cells tend to survive longer compared to those without DC (56). The study indicates that biliary tract cancer patients with myeloid dendritic cells have a more hopeful prognosis for treatment. DCs capable of initiating specific cellular responses against tumor and infectious antigens within the context of humoral immunity (57). The antigen presentation molecule HLA-DR expressed on myeloid dendritic cells plays a pivotal role in immune response, HLA-DR is a crucial antigen presentation molecule.Myeloid DC expression of HLA-DR DCs enhances their antigen presentation capacity, facilitating the immune system’s response to infections and diseases.In addition,basophil% CD33dim HLA DR - CD66b -CD33 on CD33br HLA DR+ and CD33 on CD33br HLA DR+ are negatively correlated with biliary cancer, while CD80 on myoid DC is positively correlated with biliary cancer. The research on these immune phenotypes is not very clear. Moreover, HSC AC in Myeloid cells is a risk factor for biliary cancer. Evidence suggests that cancer-associated fibroblasts (CAFs), derived from hepatic stellate cells (HSCs), facilitate the growth of intrahepatic cholangiocarcinoma by releasing hepatocyte growth factor via direct interaction in the HSC-CAFa-tumor pathway, thus activating the tumor-expressed MET pathway (58).

Our study’s strength lies in being the first to conduct MR analysis on the correlation between Immunotherapy and biliary tract malignancies. The results, Incorporating genetic factors and GWAS data, underwent rigorous assessments for horizontal pleiotropy and heterogeneity, thereby reducing confounding factors’ interference. We identified 26 immune cell phenotypes associated with biliary tract cancer, providing novel insights and avenues for exploring treatments, potential targets, and prognosis in biliary tract cancer research. However, In the aggregated data of GWAS, Analyzing overarching determinants such as age and gender in segmented form is impractical due to inherent limitations. Additionally, Our study is limited in its scope to the European population, thereby restricting the generalizability of findings to broader demographic cohorts. GWAS data from different populations or regions may exhibit significant disparities, leading to variations in the distribution and impact of various overarching determinants, including age and gender, across different demographic groups. And, we only explored the causal correlation between immunophenotypes and biliary tract malignancies, without delving into their mechanisms.In future research, our aim is to significantly increase the sample size and broaden the scope to include diverse populations. This will enable us to offer more robust theoretical support for understanding the mechanisms linking immunophenotypes and biliary tract malignancies.

## Conclusion

This study elucidates the causal relationship between immune cell phenotypes and biliary tract cancer, identifying 26 immune cell phenotypes associated with biliary tract cancer. Specifically, it highlights the association of B cells expressing CD20 and BAFF-R with reduced biliary tract cancer risk, while CD4RA+CD4+ T cells exhibit anti-tumor effects in biliary tract cancer. Conversely, Certain immunophenotypes of regulatory T cells that inhibit immune inflammation serve as protective factors against biliary tract cancer.Additionally, myeloid DCs show a negative correlation with biliary tract cancer, whereas HSC AC in Myeloid cells promotes biliary tract cancer tumor development. These discoveries offer valuable comprehension of Potential pathways and therapeutic targets for understanding and studying biliary tract cancer. A comprehensive understanding of the intricate pathways involved and potential intervention points is crucial for advancing research in this area.

## Data availability statement

The original contributions presented in the study are included in the article/**Supplementary Material**. Further inquiries can be directed to the corresponding authors.

## Ethics statement

The studies involving humans were approved by The First People’s Hospital of Yunnan Province. The studies were conducted in accordance with the local legislation and institutional requirements. The human samples used in this study were acquired from primarily isolated as part of your previous study for which ethical approval was obtained. Written informed consent for participation was not required from the participants or the participants’ legal guardians/next of kin in accordance with the national legislation and institutional requirements. Written informed consent was obtained from the individual(s) for the publication of any potentially identifiable images or data included in this article.

## Author contributions

YH: Writing – original draft, Data curation, Formal analysis, Investigation, Methodology, Project administration, Software, Supervision, Validation, Writing – review & editing. KW: Data curation, Formal analysis, Investigation, Methodology, Project administration, Software, Supervision, Validation, Writing – original draft, Writing – review & editing. YC: Formal analysis, Investigation, Methodology, Project administration, Writing – review & editing. YJ: Investigation, Methodology, Project administration, Software, Supervision, Validation, Writing – review & editing. QG: Data curation, Investigation, Project administration, Resources, Supervision, Validation, Visualization, Writing – review & editing. HT: Conceptualization, Formal analysis, Funding acquisition, Methodology, Project administration, Resources, Supervision, Validation, Visualization, Writing – review & editing.
