# Supplementary figures and images for: Causal relationship between immune cell phenotypes and risk of biliary tract cancer: evidence from Mendelian randomization analysis
Source: Front Immunol. 2024 Jul 10;15:1430551. doi: 10.3389/fimmu.2024.1430551 (PMC11266158; doi:10.3389/fimmu.2024.1430551)

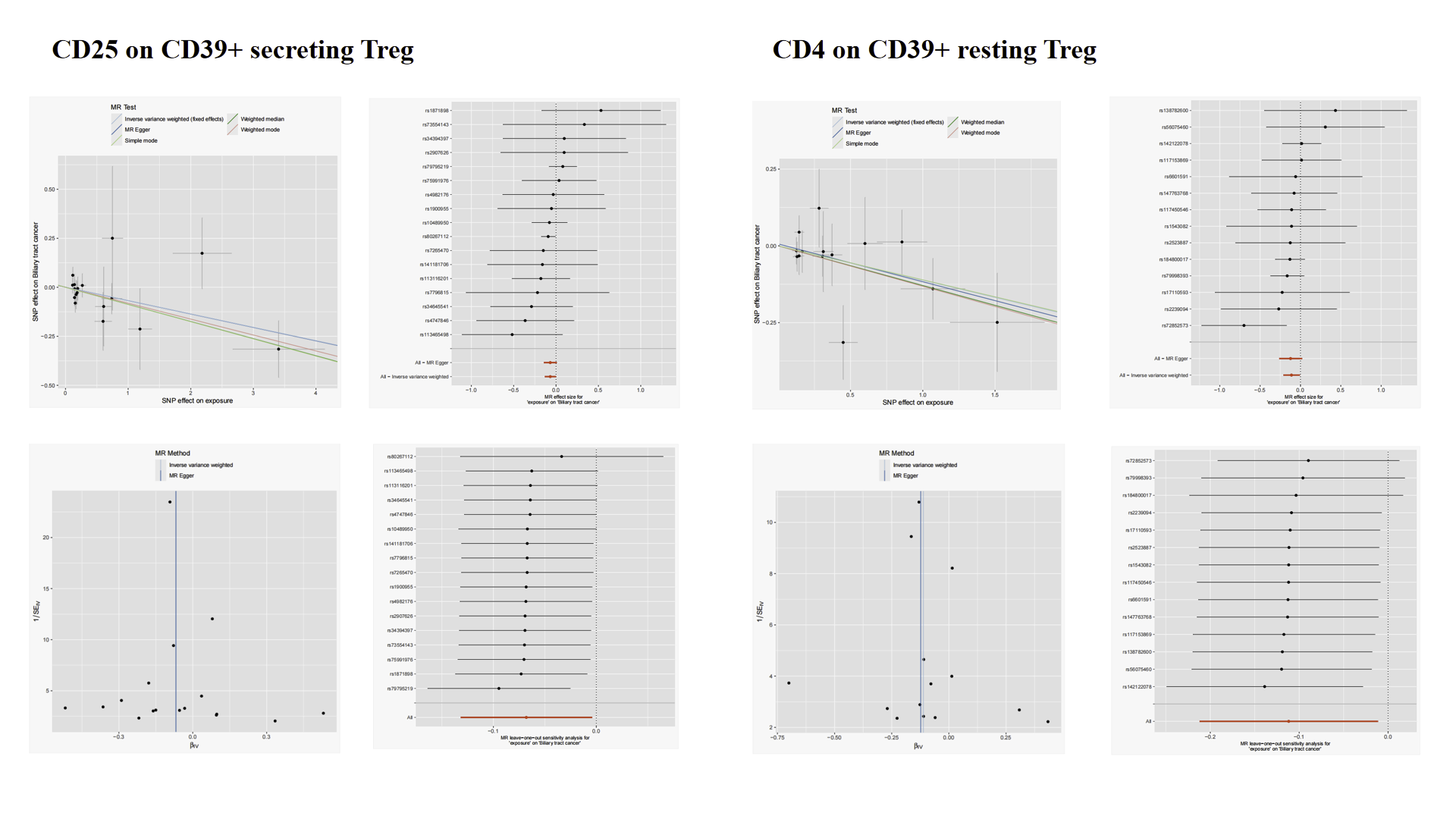

Supplement: Supplementary file 1 [file DataSheet_1.zip › Supplementary Image 1.TIFF]

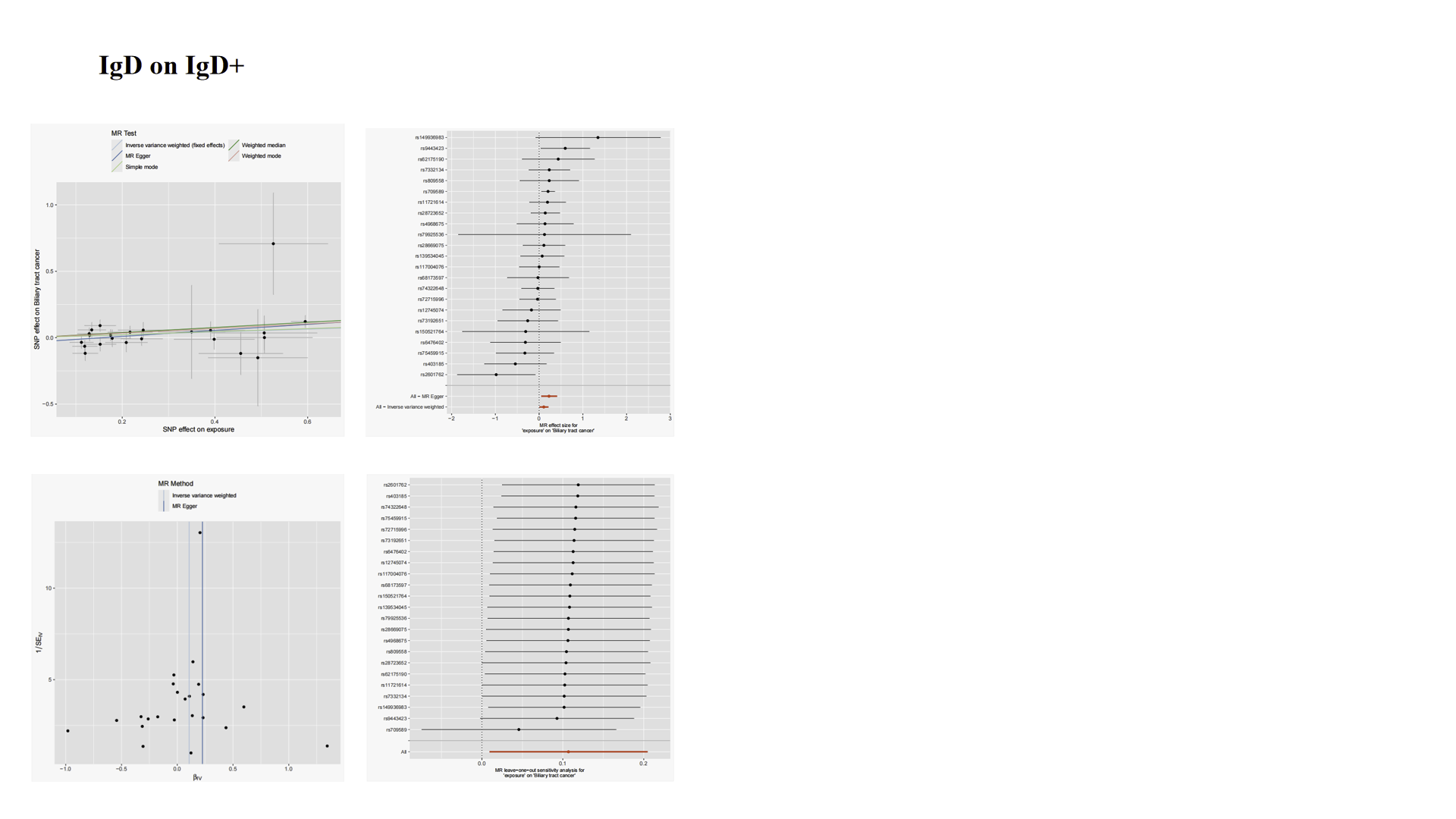

Supplement: Supplementary file 1 [file DataSheet_1.zip › Supplementary Image 10.TIFF]

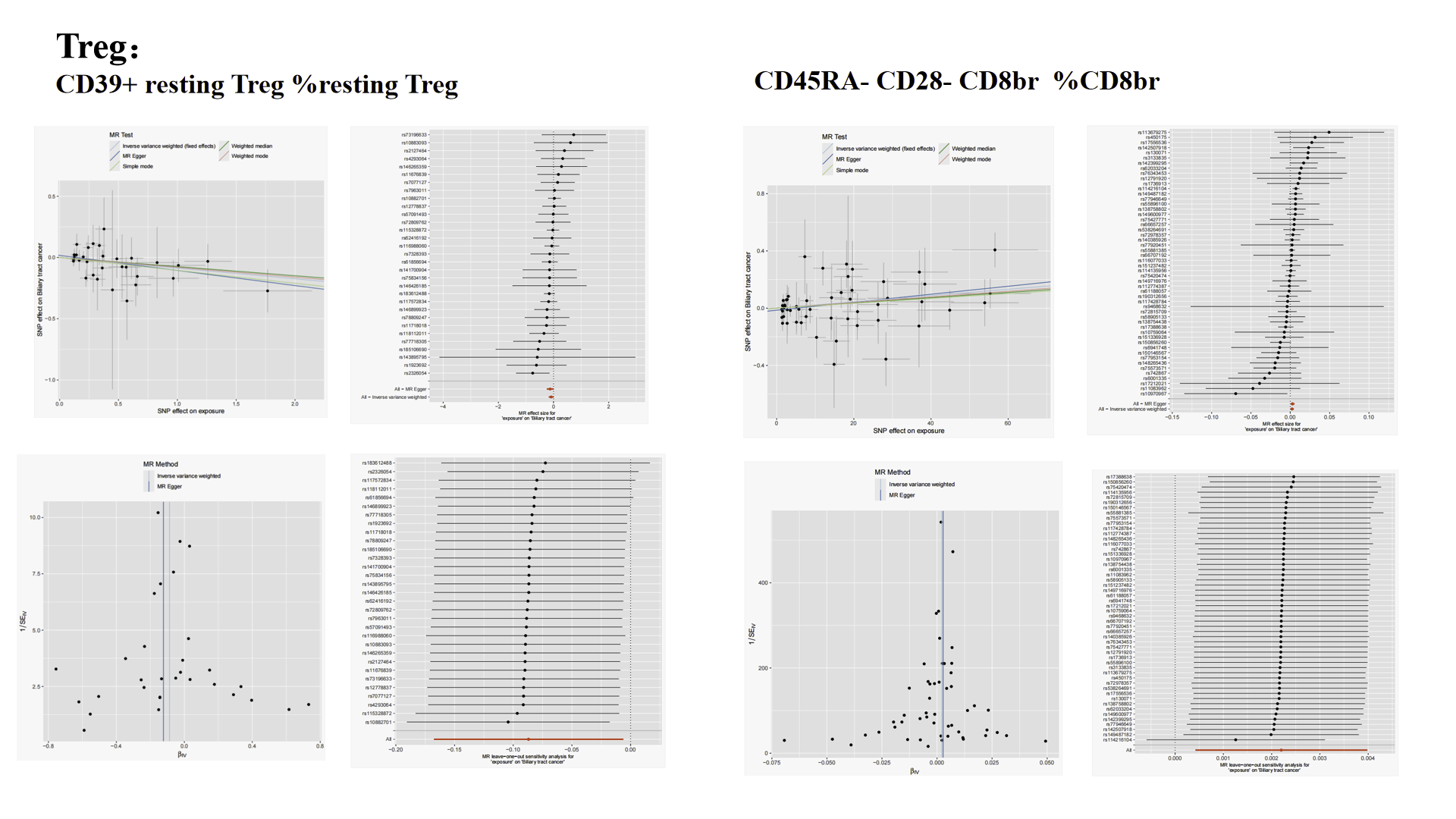

Supplement: Supplementary file 1 [file DataSheet_1.zip › Supplementary Image 11.TIFF]

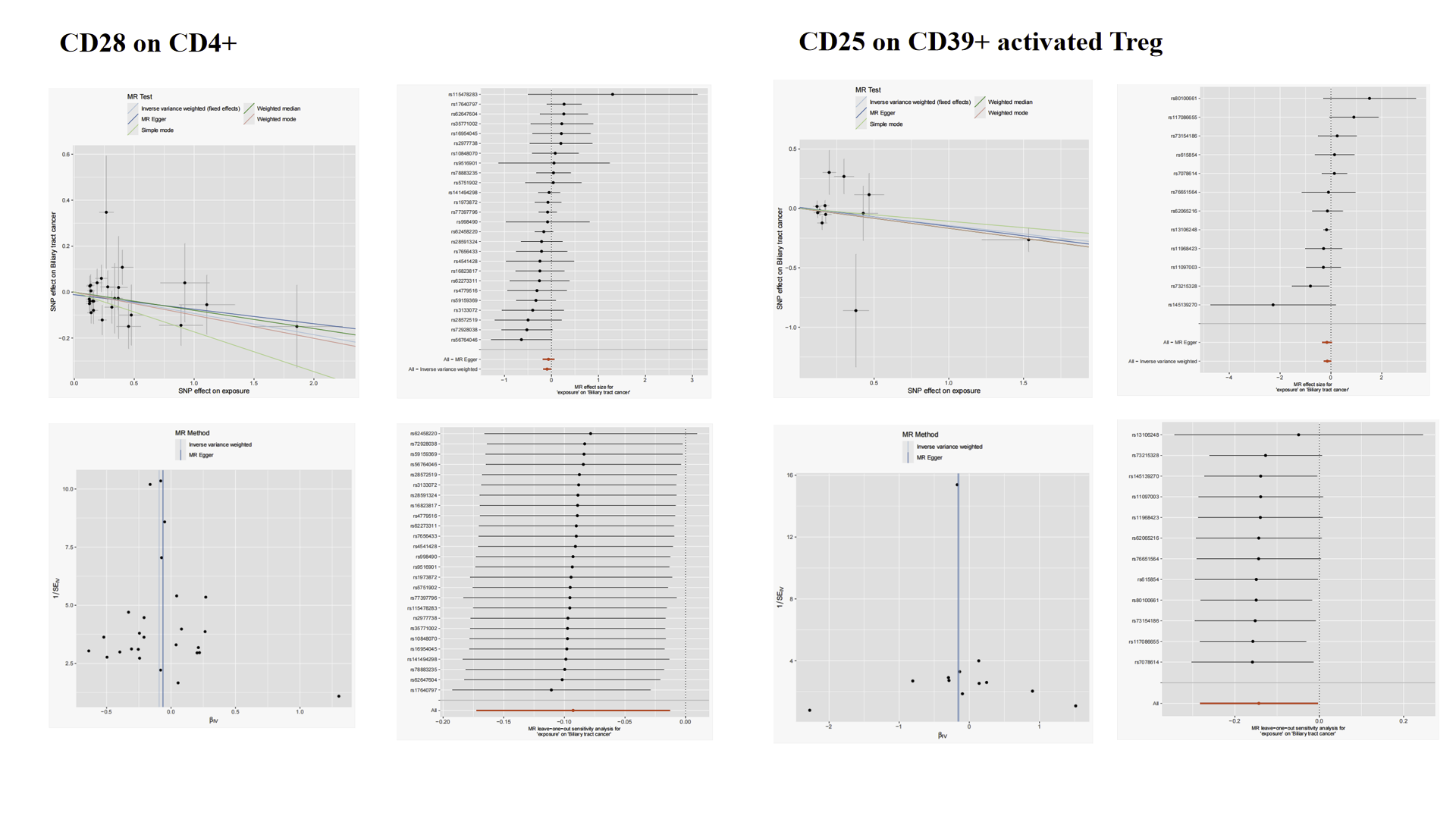

Supplement: Supplementary file 1 [file DataSheet_1.zip › Supplementary Image 12.TIFF]

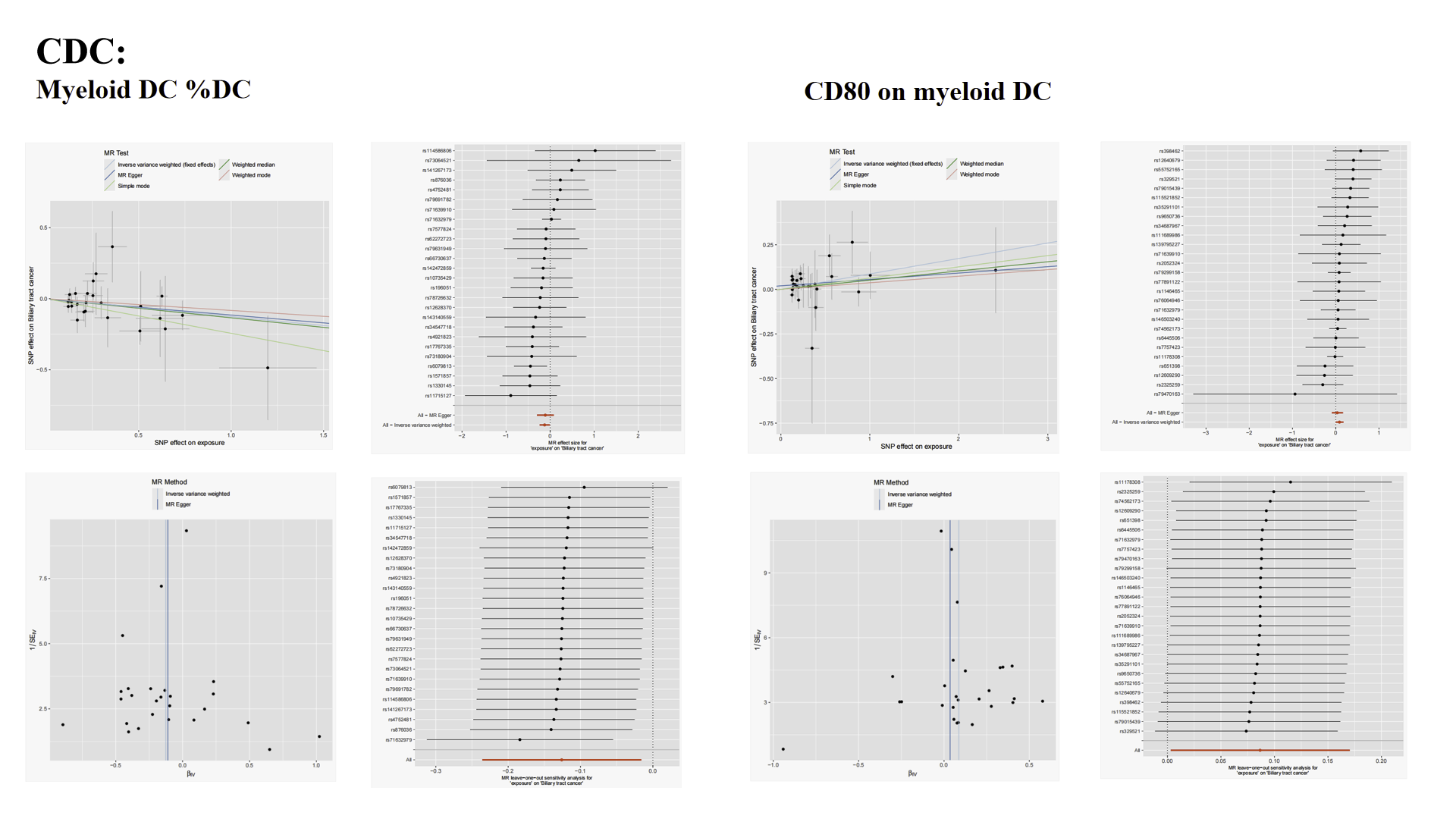

Supplement: Supplementary file 1 [file DataSheet_1.zip › Supplementary Image 2.TIFF]

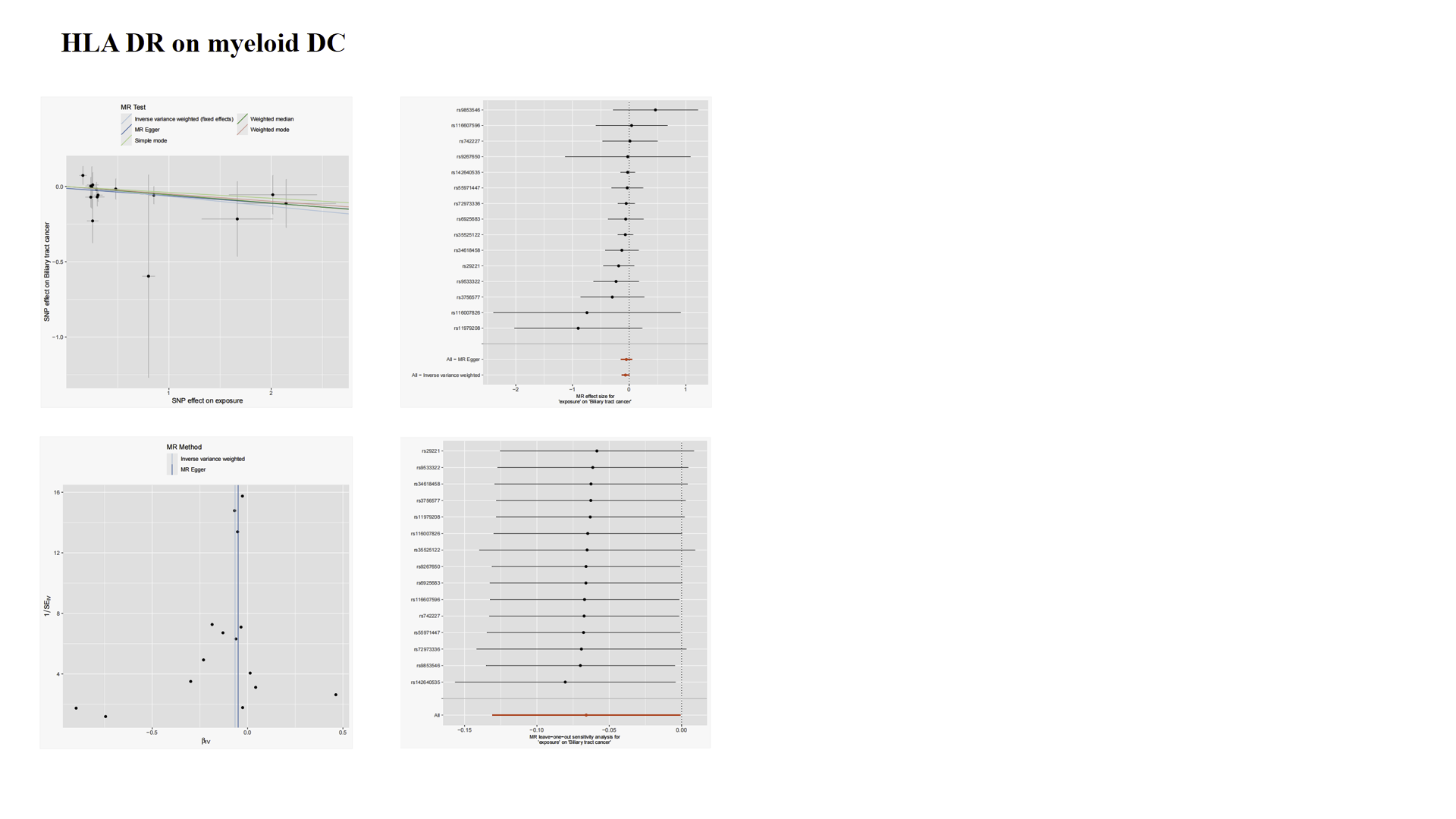

Supplement: Supplementary file 1 [file DataSheet_1.zip › Supplementary Image 3.TIFF]

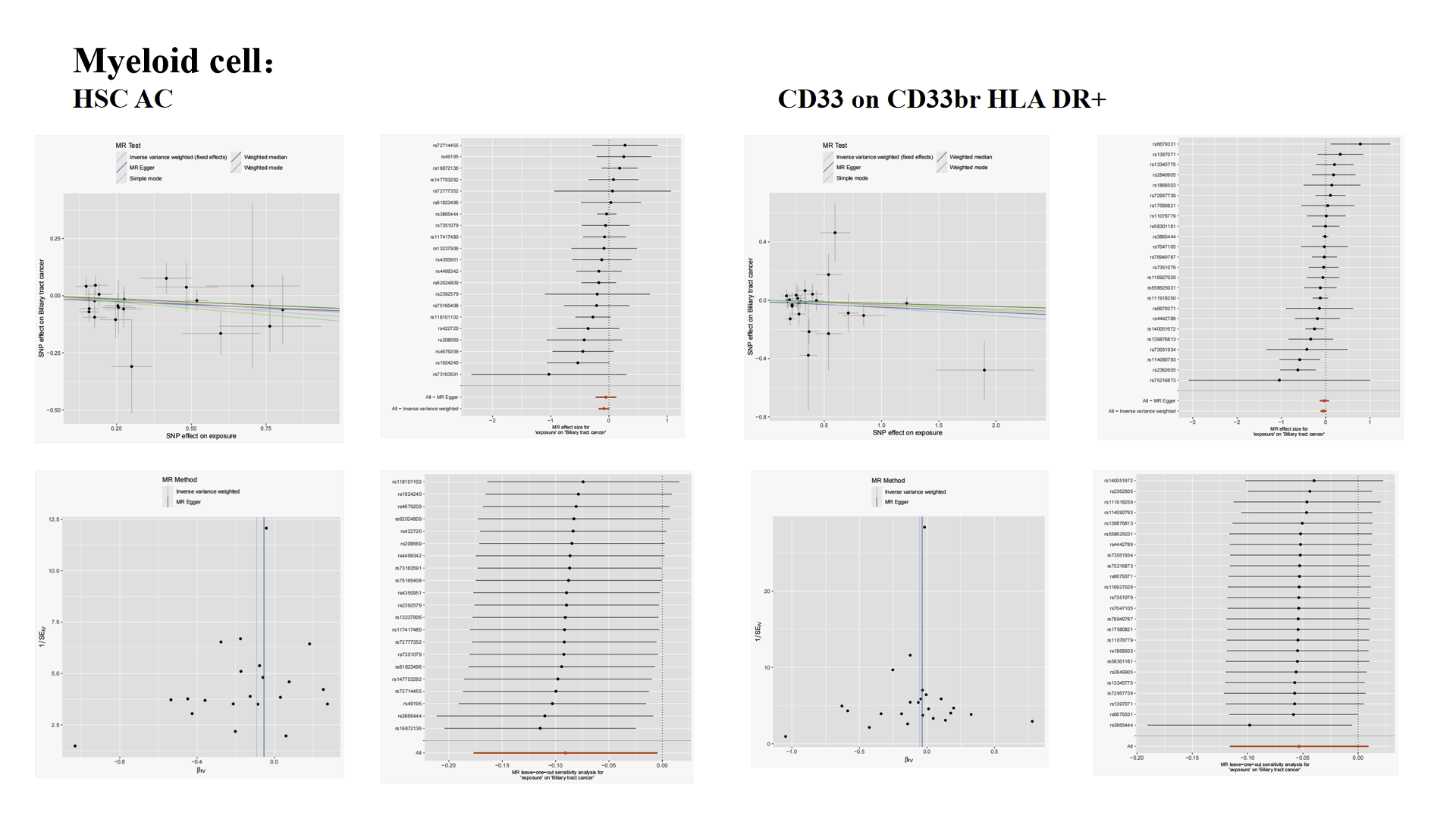

Supplement: Supplementary file 1 [file DataSheet_1.zip › Supplementary Image 4.TIFF]

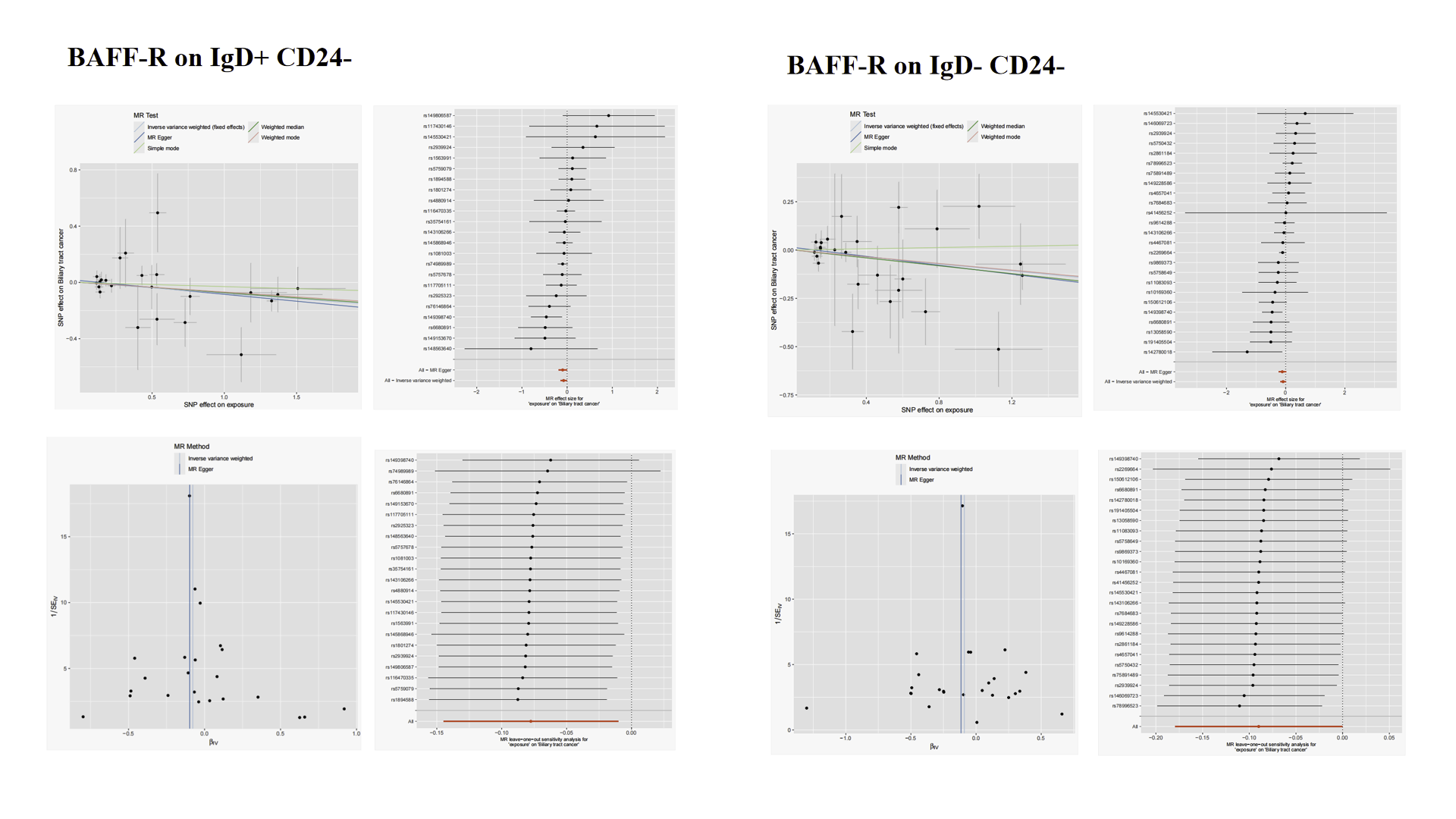

Supplement: Supplementary file 1 [file DataSheet_1.zip › Supplementary Image 5.TIFF]

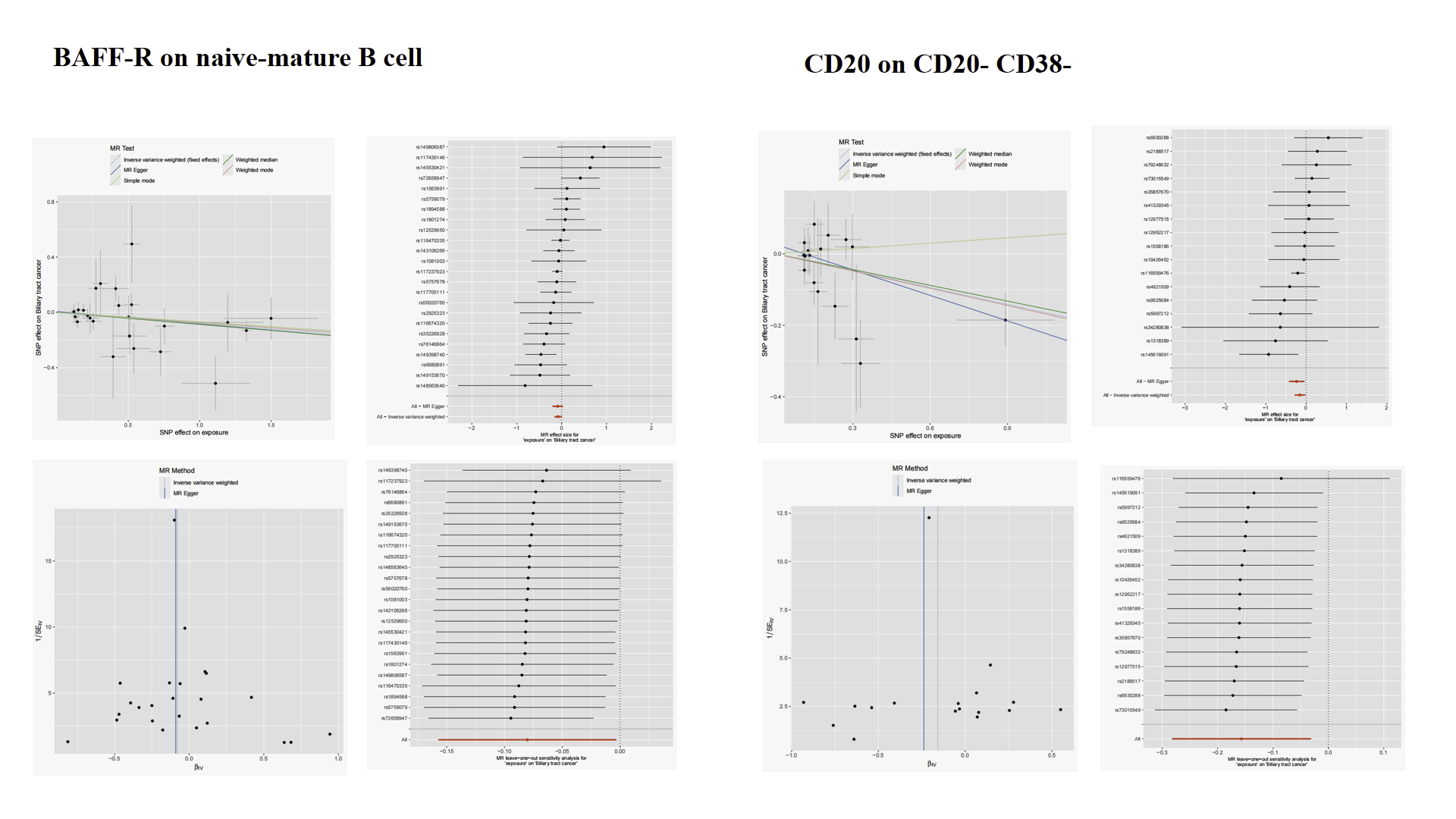

Supplement: Supplementary file 1 [file DataSheet_1.zip › Supplementary Image 6.TIFF]

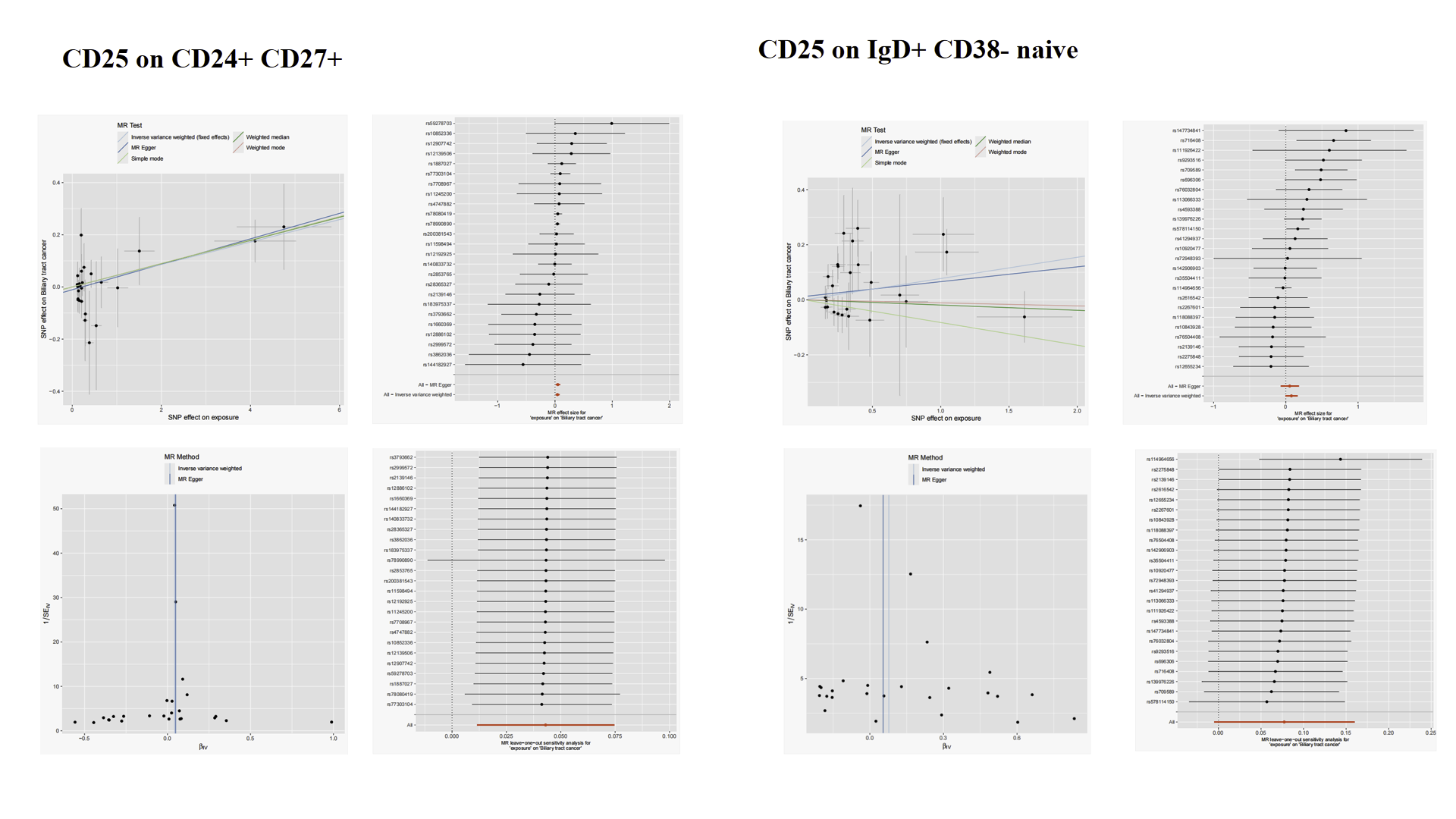

Supplement: Supplementary file 1 [file DataSheet_1.zip › Supplementary Image 7.TIFF]

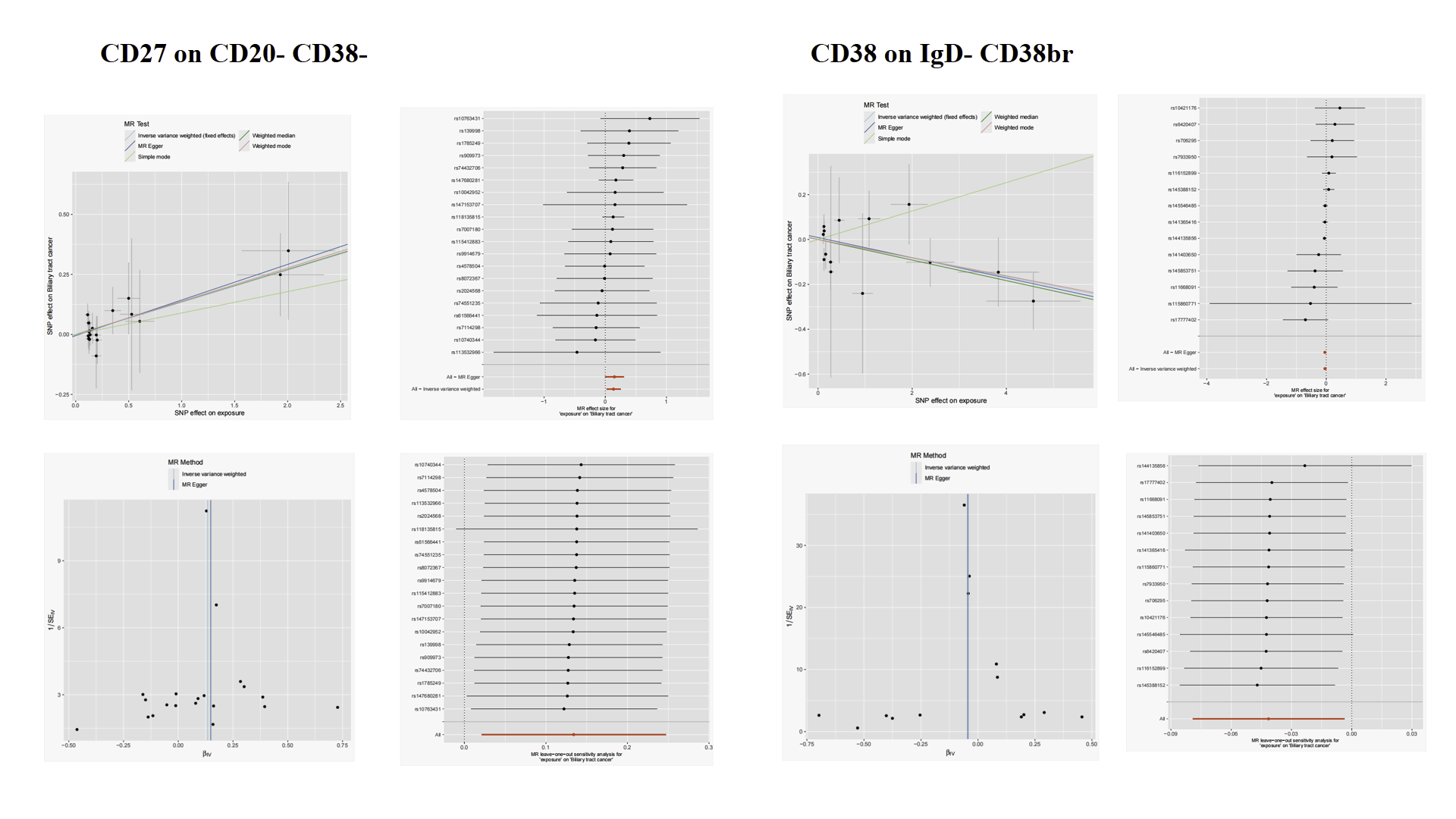

Supplement: Supplementary file 1 [file DataSheet_1.zip › Supplementary Image 8.TIFF]

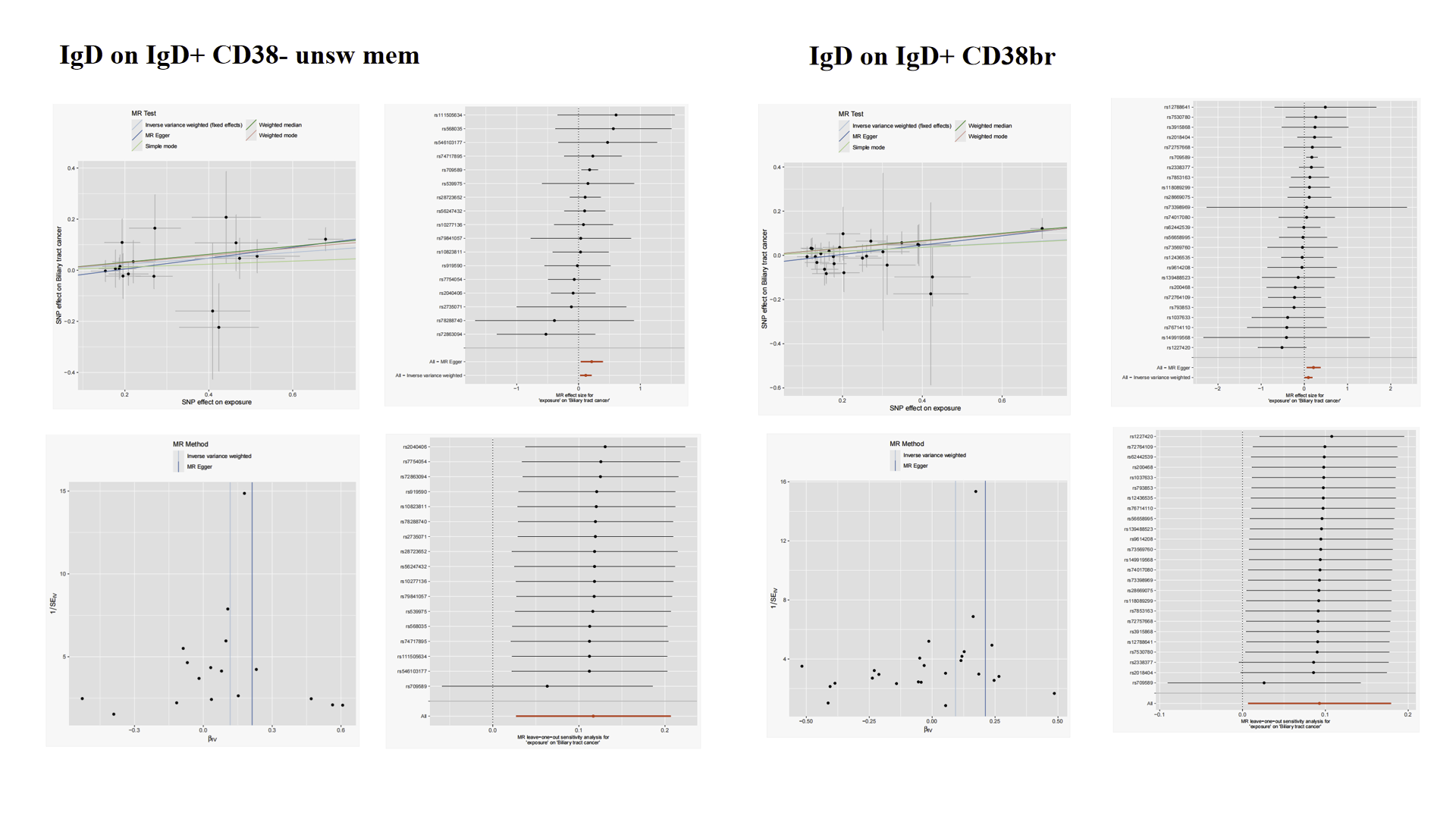

Supplement: Supplementary file 1 [file DataSheet_1.zip › Supplementary Image 9.TIFF]
